# *Klebsiella pneumoniae* with carbapenemases: high prevalence of sequence type 307 with *bla*_OXA181_ in South African community hospitals

**DOI:** 10.1007/s10096-024-04947-z

**Published:** 2024-09-17

**Authors:** Kafilat Taiwo Salvador-Oke, Johann D. D. Pitout, Gisele Peirano, Kathy-Anne Strydom, Chanel Kingsburgh, Marthie M. Ehlers, Marleen M. Kock

**Affiliations:** 1 Department of Medical Microbiology, Faculty of Health Sciences, University of Pretoria, Pretoria, South Africa; 2 Department of Pathology and Laboratory Medicine, University of Calgary, Calgary, Canada; 3 Alberta Precision Laboratories, Calgary, Canada; 4 Department of Medical Microbiology, Tshwane Academic Division, National Health Laboratory Service, Pretoria, South Africa; 5 Ampath National Reference Laboratory, Centurion, Pretoria, South Africa

**Keywords:** Carbapenemase-producing *Klebsiella pneumoniae*, High-risk clone ST307, IncX3 plasmid, *bla*_OXA-181_, Lower and middle-Income countries

## Abstract

This study investigated the molecular characteristics of urinary carbapenemase-producing *Klebsiella pneumoniae* isolates (*n* = 194) in Gauteng, South Africa, using simple, cost-effective PCR methodologies. Extensively drug resistant (XDR) ST307 with *bla*_OXA-181_ on IncX3 plasmids was endemic in Gauteng community hospitals leaving limited options for treating in- and outpatient urinary tract infections. High-level ceftazidime/avibactam resistance was detected among isolates harbouring *bla*_OXA-48-like_ including *bla*_OXA-181_. These findings highlighted the need for genomic methodologies suitable for lower- and middle-income countries to track XDR clones and plasmids in community hospitals. Such results will aid with treatment and stewardship strategies.

**Supplementary Information:**

The online version contains supplementary material available at 10.1007/s10096-024-04947-z.

## Introduction

The spread of antimicrobial resistance (AMR) genes within or between bacterial populations, is due to the persistence of successful multidrug resistant (MDR) clones and/or the movement of AMR genes within and between diverse strains [1]. Successful clones (also known as “high-risk” clones) are found among various bacterial pathogens especially within *Pseudomonas aeruginosa,* and the *Enterobacterales* [2]. *Klebsiella pneumoniae* has a panmictic population structure that consists of certain successful clones among non-MDR populations (e.g. sequence type (ST)-23, ST86), while different high-risk clones are found within MDR populations (e.g. ST147, ST258, and ST307) [3].

Carbapenemases inactivate most available β-lactam antimicrobials [4]. They belong to the Ambler classes A, B and D. Class A and D are serine β-lactamases (i.e. KPCs, OXA-48-like), while class B are metallo-β-lactamases (MBLs) i.e. NDMs, VIMs and IMPs [5].

A previous South African study showed high frequency of ST307 among carbapenemase-producing *K. pneumoniae* in a tertiary care center [6]. We determined the prevalence and molecular characteristics of urinary carbapenemase-producing *K. pneumoniae* isolates obtained from community hospitals in Gauteng, South Africa, using simple, cost-effective PCR methodologies. Our results showed the high prevalence of extensively drug resistant (XDR) ST307 with *bla*_OXA-181_ on IncX3 plasmids that will aid with treatment and control strategies of patients within community hospitals.

## Materials and methods

Non-duplicate carbapenem (i.e. ertapenem, and/or meropenem, and/or imipenem) resistant *K. pneumoniae* urinary isolates (*n* = 194) were collected from 71 community hospitals in Gauteng, South Africa between Feb 2021, and May 2022. Isolates were identified using matrix assisted laser desorption ionization-time of flight mass spectrometry [MALDI-TOF MS, Bruker Daltonics, United States] and antimicrobial susceptibility testing was performed by disk diffusion method using the European Committee on Antimicrobial Susceptibility Testing (EUCAST) guidelines [7]. Standard definitions were used for MDR, XDR, and pandrug resistant (PDR) bacteria [8].

Carbapenemase genes (i.e. *bla*_IMP_, *bla*_KPC_, *bla*_NDM_, *bla*_OXA-48-like_, *bla*_VIM_) were detected using PCR as previously described [9, 10]. PCRs for ST307, IncX3 plasmids, OXA-181-IS*3000* and OXA-48-IS*1999* were performed as previously described [11]. The rapid polymyxin Nordmann/Poirel test was used to screen for polymyxin resistance [12] and colistin microbroth dilution were performed on positive isolates, using EUCAST guidelines [13]. PCR for plasmid-mediated colistin resistant (*mcr*) genes was performed on colistin resistant isolates using previously published methods [14–16].

Patients’ data were processed using SPSS statistical software version 28.0.0 (SPSS Incorporation, Chicago, IL, US). Fisher’s exact test was used for comparison of categorical data. One-sample proportion test was used to estimate the proportions of ST307 and non-ST307 among inpatient and outpatient populations. Two-sample proportion test was used to compare the molecular profiles of ST307 and non-ST307. Results were considered as statistically significant if *p* < 0.05 was achieved at a 95% confident interval.

## Results

Carbapenem-resistant *K. pneumoniae* were collected from inpatients (*n* = 166) and outpatients (*n* = 28) throughout the Gauteng province. The study population comprised of 101 (52%) male patients and 93 (48%) female patients with a mean age of 61 ± 17 years (range 21 days and 98 years) (Table 1). Most of the isolates (84%) were XDR followed by MDR (12%) and PDR (4%) (Table 1). Resistance to colistin and ceftazidime/avibactam were 7% and 19% respectively. Colistin resistant isolates (*n* = 14) were negative for *mcr* genes. Some ceftazidime/avibactam resistant isolates (20/37 [54%]) contained MBLs. Of interest was that 16/37 (43%) of ceftazidime/avibactam resistant isolates were positive for *bla*_OXA-48-like_ including two (5%) *bla*_OXA-181_. In addition, one (3%) ceftazidime/avibactam resistant isolate harboured *bla*_KPC_. More detail in supplementary File S1:Table 1.

**Table 1:** Characteristics of urinary carbapenemase-producing *Klebsiella pneumoniae* isolates from community hospital, Gauteng, South Africa

Characteristics	ST307 (*n* = 95)	Non-ST307 (*n* = 99)	Total (*n* = 194)	*p*-value
Patient details:				
Mean age	61 yrs	61 yrs	-	0.951
Female	45 (47%)	48 (48%)	93 (48%)	0.014*
Male	50 (53%)	51 (52%)	101 (52%)	-
Inpatient	81 (85%)	85 (86%)	166 (86%)	0.007*
Outpatient	14 (15%)	14 (14%)	28 (14%)	0.007*
Not susceptible (intermediate or resistant):				
IMP	61 (64%)	64 (65%)	125 (64%)	-
ETP	94 (99%)	98 (99%)	192 (99%)	-
MEM	79 (83%)	66 (67%)	145 (75%)	-
TZP	95 (100%)	99 (100%)	194 (100%)	-
CXM	95 (100%)	99 (100%)	194 (100%)	-
AMC	95 (100%)	99 (100%)	194 (100%)	-
CRO	95 (100%)	97 (98%)	192 (99%)	-
CAZ	94 (99%)	96 (97%)	190 (98%)	-
CZA	10 (11%)	27 (27%)	37 (19%)	-
FEP	93 (98%)	92 (93%)	185 (95%)	-
GEN	78 (82%)	84 (85%)	162 (84%)	-
AMK	55 (58%)	61 (62%)	116 (60%)	-
CIP	94 (99%)	93 (94%)	187 (96%)	-
SXT	92 (97%)	88 (89%)	180 (93%)	-
CST	8 (8%)	6 (6%)	14 (7%)	0.587
Resistance profiles:				0.259
MDR	12 (13%)	12 (12%)	24 (12%)	-
XDR	78 (82%)	85 (86%)	163 (84%)	-
PDR	5 (5%)	2 (2%)	7 (4%)	-
Carbapenemase genes:				
OXA-181 only	58 (61%)	38 (38%)	96 (49%)	0.002*
OXA-48 only	36 (38%)	55 (58%)	91 (47%)	0.369
NDM only	0 (0%)	3 (20%)	3 (2%)	0.012*
KPC only	2 (2%)	2 (2%)	4 (2%)	1.000
VIM only	1 (1%)	1 (1%)	2 (1%)	1.000
OXA-181 + NDM	4 (4%)	6 (6%)	10 (5%)	-
OXA-181 + KPC	1 (1%)	0 (0%)	1 (0.5%)	-
OXA-181 + VIM	1 (1%)	0 (0%)	1 (0.5%)	-
OXA-48 + NDM	3 (3%)	11 (11%)	14 (7%)	-
IncX3 plasmids	60 (63%)	39 (39%)	99 (51%)	0.001*

*Statistically significant *p*-value = 0.05 or lower. Multidrug resistant (MDR), Extensively drug resistant (XDR), Pan-drug resistant (PDR) Ampicillin (AMP), Amoxicillin/Clavulanate (AMC), Piperacillin/tazobactam (TZP), Cefuroxime (CXM), Ceftriaxone (CRO), Ceftazidime (CAZ), Ceftazidime/Avibactam (CZA), Cefepime (FEP), Ertapenem (ETP), Imipenem (IPM), Meropenem (MEM), Amikacin (AMK), Gentamicin (GEN), Ciprofloxacin (CIP), Co-trimoxazole (SXT), Colistin (CST)

All the carbapenem-resistant *K. pneumoniae* harboured carbapenemase genes (Table 1): most (*n* = 96) contained *bla*_OXA-181_ followed by *bla*_OXA-48_ (*n* = 91), *bla*_NDM_ (*n* = 27), *bla*_KPC_ (*n *= 4), and *bla*_VIM_ (*n* = 2). Twenty-six isolates were positive for two carbapenemases e.g. *n* = 10 with *bla*_OXA-181_ + *bla*_NDM_, *n* = 14 with *bla*_OXA-48_ + *bla*_NDM_, one each with *bla*_OXA-181_ + *bla*_KPC_ and *bla*_OXA-181_ + *bla*_VIM_ respectively.

Nearly half of carbapenemase-producing *K. pneumoniae* (i.e. 49% (95/194) belonged to ST307 (Table 1). Most of urines with ST307 were submitted from inpatients (*n* = 81), while 14 urines were submitted from outpatients (Table 1). There were statistically significant differences between ST307 and non-ST307 *K. pneumoniae* with patient characteristics but no significance observed with AMR profiles (Table 1). OXA-181 genes with IS3000, and IncX3 plasmids were significantly more common among ST307 than non-ST307 *K. pneumoniae*, while *bla*_NDM_ and *bla*_OXA-48_ were more common among non-ST307 isolates (Table 1). Two carbapenemases in the same isolate were detected in ST307 (*n* = 9) and non-ST307 (*n* = 17) (Table 1).

## Discussion

Lower- and middle-income countries (LMICs) bear considerable AMR burden but lack adequate genomic diagnostic tools to identify and track such bacteria including high-risk MDR clones [17]. LMIC genomic methodologies should be simple, user-friendly, and accordant with local economic constraints [17]. The identification of high-risk clones and AMR plasmids often requires sequencing, which is expensive and time consuming and not available in most LMICs [17]. This study used simple, cost-effective PCR methodologies to identify *K. pneumoniae* ST307 and IncX3 plasmids in the LMIC setting. Our results are especially suitable for endemic regions within LMICs with a high prevalences of ST307 for tracking the movement of this MDR clone across different health care systems [17].

There has been a global increase in ST307, and South Africa has been recognised as an endemic country with high prevalences (> 70%) among carbapenemase-producing isolates during hospital outbreaks [3, 6, 11]. Recently reported global rates among hospital carbapenemase-producing *K. pneumoniae* included Italy (29%; 27/94), Russia (29%; 46/159), Spain (22%; 82/377) and South Korea (27%; 12/45) [18–21]. In our study, a high prevalence of ST307 of nearly 50% was observed among carbapenemase-producing *K. pneumoniae* showing that this MDR clone is endemic in Gauteng community hospitals. Of concern is that over 80% of ST307 and non-ST307 from inpatients tested XDR leaving limited options for treating patients with urinary tract infections (UTIs).

A study from Panama included outpatients and detected six ST307 isolates among 11 (55%) carbapenem-resistant *K. pneumoniae* [22]. The frequency of ST307 among outpatient urines in our study was similar (14/28 [50%]). Few oral options were available to treat patients with community-onset UTIs due to *K. pneumoniae* ST307 and non-ST307 in the Gauteng region. This will complicate the treatment of outpatient UTIs in the Gauteng region since oral antimicrobials are the only cost-effective options available [23]. The high resistance rates among patients might be due to the excessive use of broad-spectrum antimicrobials such as ciprofloxacin for treating UTIs in South Africa [24]. Urgent stewardship intervention is required to improve compliance with treatment recommendations based on standard treatment guidelines for selecting suitable antimicrobials to treat UTIs [24].

South African antimicrobial stewardship (AMS) treatment guidelines recommended the use of colistin in combination with rifampicin or carbapenems for treating severe infections due to carbapenem-resistant *K. pneumoniae* [25]. Ceftazidime/avibactam and ceftolozane/tazobactam are last-resort antimicrobials and empirical use is discouraged for UTIs [26]. The resistance to ceftazidime/avibactam in this study was partly due to the lack of activity against MBL isolates [27]. Of special concern, was the high ceftazidime/avibactam resistance detected among non-MBL isolates (with *bla*_OXA-48-like_ including *bla*_OXA-181_)_,_ as recently reported from Egypt and India [28, 29].

High-risk MDR Gram-negative clones are not directly responsible for the movement of AMR genes but act as “hoarders and spreaders” of such genes [30]. This was illustrated by a previous study from a tertiary care centre in Gauteng [6]. *Klebsiella pneumoniae* ST307 with *bla*_OXA-181_ harboured on 51kb IncX3 plasmids, was introduced during September 2015 [6]. The ST307 spread throughout the hospital causing severe nosocomial outbreaks during 2015. The authors showed that IncX3 plasmids containing *bla*_OXA-181_ was introduced by ST307 and then transferred to other *K. pneumoniae* STs over time [6]. Nearly 40% of non-ST307 *K. pneumoniae* with carbapenemases in our study contained IncX3 plasmids with *bla*_OXA-181_, suggesting that IncX3 plasmids with *bla*_OXA-181_ have also found their way to non-ST307 *K. pneumoniae* isolates in community hospitals.

The laboratory detection of *Enterobacterales* with OXA-48-like carbapenemases remains challenging for some clinical laboratories [31]. The OXA-48-like producing bacteria can test sensitive to carbapenems, especially among isolates without extended-spectrum β-lactamases or AmpC β-lactamases [32, 33]. As single screening agents, meropenem provides the best balance between sensitivity and specificity; ertapenem has a high sensitivity but lacks specificity, while imipenem does not reliably distinguish between wild-type isolates in species such as *Proteus* spp., *Providencia* spp., and *Morganella morganii* [31]. Over 95% of *K. pneumoniae* from this study tested not susceptible to ertapenem, while 75% tested not susceptible to meropenem and only 64% to imipenem. This suggest that clinical laboratories in endemic regions should as a minimum include ertapenem as a screening agent for *Enterobacterales* with OXA-48-like carbapenemases.

In summary, the *K. pneumoniae* XDR high-risk clone ST307 is endemic in Gauteng community hospitals and was the main driver of several carbapenemase genes, particularly *bla*_OXA-181_. The *bla*_OXA-181_ is present on epidemic IncX3 plasmids that were prevalent among the *K. pneumoniae* isolates (ST307 and non-ST307) circulating among different patient groups in Gauteng, South Africa. High-level ceftazidime/avibactam resistance was detected among isolates with *bla*_OXA-48-like_ including *bla*_OXA-181_. These findings highlighted the need for genomic surveillance of carbapenem-resistant *K. pneumoniae* high-risk clones and associated plasmids using methodologies suitable for LMICs to track XDR clones and plasmids in community hospitals. Such results will aid with treatment and stewardship strategies in LMICs that will ultimately improves outcomes of patients with UTIs.

## Supplementary Information

Below is the link to the electronic supplementary material.

Supplementary file1 (PDF 697 KB)

## Data Availability

No datasets were generated or analysed during the current study.
