# Supplementary material for: Klebsiella pneumoniae with carbapenemases: high prevalence of sequence type 307 with blaOXA181 in South African community hospitals
Source: Eur J Clin Microbiol Infect Dis. 2024 Sep 17;43(11):2239–44. doi: 10.1007/s10096-024-04947-z (PMC11534848; doi:10.1007/s10096-024-04947-z)
Supplement: Supplementary file 1 — Supplementary file1 (PDF 697 KB) [file 10096_2024_4947_MOESM1_ESM.pdf]

Klebsiella pneumoniae with carbapenemases: high prevalence of sequence type 307 with bla_{OXA181} in South African community hospitals

Kafilat Taiwo Salvador-Oke[1]. Johann DD Pitout[1, 2, 3]. Gisele Peirano[2, 3]. Kathy-Anne Strydom[4, 5]. Chanel Kingsburgh[4, 5]. Marthie M Ehlers[1, 4].

Marleen M Kock[1,4]

Supplementary File S1: Table 1 Carbapenemase-producing *Klebsiella pneumoniae* isolates profile

			Antimicrobials																Carbapenemase genes					
Isolates	ST307	IncX3	AMP	AMC	TZP	CXM	CRO	CAZ	CZA	FEP	ETP	IPM	MEM	AMK	GEN	CIP	SXT	CST	bla_{KPC}	bla_{NDM}	$bla_{OXA\text{-}48\text{-}like}$	$bla_{OXA\text{-}181}$	bla_{VIM}	RES Level
UCRKP1	no	no	R	R	R	R	R	R	ND	R	R	S	S	S	S	R	R	S	neg	neg	pos	pos	neg	XDR
UCRKP2	no	yes	R	R	R	R	R	R	ND	R	R	R	R	R	R	R	R	S	neg	neg	pos	pos	neg	XDR
UCRKP3	no	yes	R	R	R	R	R	R	S	R	R	R	R	R	R	R	R	S	neg	neg	pos	pos	neg	XDR
UCRKP4	no	yes	R	R	R	R	R	R	S	R	R	S	S	S	R	R	R	S	neg	neg	pos	pos	neg	MDR
UCRKP5	yes	yes	R	R	R	R	R	R	ND	R	R	I	R	S	R	R	R	S	neg	neg	pos	pos	neg	XDR
UCRKP6	no	no	R	R	R	R	R	R	S	R	R	R	R	R	R	R	R	S	neg	neg	pos	pos	neg	XDR
UCRKP7	yes	yes	R	R	R	R	R	R	S	R	R	I	R	S	R	R	R	S	neg	neg	pos	pos	neg	XDR
UCRKP8	no	no	R	R	R	R	R	R	ND	R	R	S	S	S	S	R	S	S	neg	neg	pos	neg	neg	MDR
UCRKP9	yes	yes	R	R	R	R	R	R	ND	R	R	I	R	R	R	R	R	S	neg	neg	pos	pos	neg	XDR
UCRKP10	yes	no	R	R	R	R	R	R	S	R	R	R	R	R	R	R	R	S	neg	neg	pos	neg	neg	XDR
UCRKP12	yes	yes	R	R	R	R	R	R	ND	R	R	S	R	I	S	R	R	R	neg	neg	pos	pos	neg	PDR
UCRKP13	yes	yes	R	R	R	R	R	R	S	R	R	R	R	I	R	R	R	S	neg	neg	pos	pos	neg	XDR
UCRKP15	no	yes	R	R	R	R	R	R	ND	R	R	S	S	S	S	R	ND	S	neg	neg	pos	pos	neg	MDR
UCRKP17	no	no	R	R	R	R	R	R	S	R	R	S	S	S	R	R	R	S	neg	neg	pos	pos	neg	XDR
UCRKP18	yes	yes	R	R	R	R	R	R	ND	R	R	S	I	R	R	R	R	S	neg	neg	pos	pos	neg	XDR
UCRKP19	no	no	R	R	R	R	R	R	ND	R	R	I	S	S	S	R	R	S	neg	neg	pos	pos	neg	XDR

UCRKP20	no	no	R	R	R	R	ND	R	S	ND	R	S	S	S	S	S	S	S	neg	neg	pos	neg	neg	MDR
UCRKP21	no	yes	R	R	R	R	R	R	ND	R	R	R	R	R	R	R	R	S	neg	neg	pos	pos	neg	XDR
UCRKP22	no	yes	R	R	R	R	R	R	ND	ND	R	R	R	R	R	R	R	S	neg	neg	pos	pos	neg	XDR
UCRKP23	no	yes	R	R	R	R	R	R	R	R	R	I	R	R	R	R	R	S	neg	pos	pos	pos	neg	XDR
UCRKP24	no	no	R	R	R	R	ND	R	R	R	R	R	R	R	S	R	R	S	neg	pos	pos	neg	neg	XDR
UCRKP25	no	no	R	R	R	R	R	R	R	R	R	R	R	R	R	R	R	S	neg	pos	pos	neg	neg	XDR
UCRKP26	yes	yes	R	R	R	R	R	R	S	R	R	I	R	R	R	R	R	S	neg	neg	pos	pos	neg	XDR
UCRKP29	no	yes	R	R	R	R	R	R	S	R	R	R	R	R	R	R	R	S	neg	neg	pos	pos	neg	XDR
UCRKP30	no	no	R	R	R	R	R	R	ND	R	R	S	I	R	R	R	R	S	neg	pos	pos	neg	neg	XDR
UCRKP31	no	no	R	R	R	R	R	R	ND	R	R	S	S	R	S	R	R	S	neg	neg	pos	neg	neg	XDR
UCRKP32	no	yes	R	R	R	R	R	R	ND	ND	R	I	R	S	R	R	R	S	neg	neg	pos	pos	neg	XDR
UCRKP33	yes	yes	R	R	R	R	R	R	S	R	R	S	S	R	R	R	R	S	neg	neg	pos	pos	neg	XDR
UCRKP34	yes	yes	R	R	R	R	R	R	S	R	R	S	R	S	R	R	R	S	neg	neg	pos	pos	neg	XDR
UCRKP35	yes	yes	R	R	R	R	R	R	ND	R	R	S	S	S	S	R	R	S	neg	neg	pos	pos	pos	XDR
UCRKP36	no	no	R	R	R	R	R	R	S	R	R	S	S	S	R	R	S	S	neg	neg	pos	pos	neg	MDR
UCRKP37	no	yes	R	R	R	R	R	R	ND	R	R	S	S	S	R	R	R	S	neg	neg	pos	pos	neg	XDR
UCRKP38	no	yes	R	R	R	R	R	R	S	R	R	S	S	S	S	S	S	S	neg	neg	pos	neg	neg	MDR
UCRKP39	no	no	R	R	R	R	R	R	S	R	R	S	S	ND	ND	ND	ND	S	neg	neg	pos	pos	neg	MDR
UCRKP40	yes	yes	R	R	R	R	R	R	S	R	R	R	R	S	R	R	R	S	neg	neg	pos	pos	neg	XDR
UCRKP42	no	yes	R	R	R	R	R	R	S	R	R	I	I	S	R	R	R	S	neg	neg	pos	neg	neg	XDR
UCRKP43	no	yes	R	R	R	R	R	R	ND	R	R	S	S	S	R	R	R	S	neg	neg	pos	pos	neg	XDR
UCRKP44	no	yes	R	R	R	R	R	S	S	S	R	S	S	S	S	S	S	S	neg	neg	pos	neg	neg	MDR
UCRKP45	no	yes	R	R	R	R	R	R	S	R	R	S	I	S	R	R	S	S	neg	neg	pos	neg	neg	MDR
UCRKP46	no	no	R	R	R	R	R	R	S	R	R	I	R	S	R	R	R	S	neg	neg	pos	neg	neg	XDR
UCRKP47	no	yes	R	R	R	R	R	R	S	R	R	S	S	S	R	R	R	S	neg	neg	pos	neg	neg	XDR
UCRKP48	no	yes	R	R	R	R	R	ND	ND	ND	R	I	S	S	R	R	R	S	neg	neg	pos	neg	neg	XDR
UCRKP49	yes	yes	R	R	R	R	R	R	ND	R	R	I	R	S	R	R	R	S	neg	neg	pos	neg	neg	XDR
UCRKP50	no	yes	R	R	R	R	R	R	ND	R	R	S	R	R	R	R	S	S	neg	neg	pos	neg	neg	XDR
UCRKP51	yes	yes	R	R	R	R	R	R	S	R	R	R	R	S	S	R	R	S	neg	neg	pos	neg	neg	MDR
UCRKP52	yes	yes	R	R	R	R	R	R	S	R	R	S	R	R	R	R	R	S	neg	neg	pos	neg	neg	XDR

UCRKP53	no	yes	R	R	R	R	R	R	S	R	R	S	S	R	R	R	R	S	neg	neg	pos	neg	neg	XDR
UCRKP54	no	no	R	R	R	R	R	R	R	R	R	I	I	R	R	R	R	S	neg	neg	pos	neg	neg	XDR
UCRKP55	no	no	R	R	R	R	R	R	ND	R	R	I	I	S	R	R	R	S	neg	neg	pos	pos	neg	XDR
UCRKP56	yes	no	R	R	R	R	R	R	S	R	R	I	R	S	S	R	R	S	neg	neg	pos	pos	neg	MDR
UCRKP57	no	yes	R	R	R	R	R	R	S	R	R	S	S	S	R	R	R	S	neg	neg	pos	neg	neg	XDR
UCRKP58	no	yes	R	R	R	R	R	R	S	R	R	R	R	S	R	R	R	S	neg	neg	pos	neg	neg	XDR
UCRKP59	yes	no	R	R	R	R	R	R	ND	R	R	R	R	S	R	R	R	S	neg	neg	pos	neg	neg	XDR
UCRKP61	no	no	R	R	R	R	R	R	R	R	R	R	R	R	R	R	R	S	neg	neg	pos	neg	neg	XDR
UCRKP62	no	no	R	R	R	R	R	R	ND	R	R	R	I	S	S	R	R	S	neg	neg	pos	pos	neg	XDR
UCRKP64	no	no	R	R	R	R	R	R	S	R	R	S	S	S	R	R	R	S	neg	neg	pos	pos	neg	XDR
UCRKP65	no	no	R	R	R	R	R	R	S	R	R	R	R	R	R	R	R	S	neg	neg	pos	neg	neg	XDR
UCRKP66	no	no	R	R	R	R	R	R	R	R	R	R	R	R	R	R	R	S	neg	pos	neg	neg	neg	XDR
UCRKP68	no	no	R	R	R	R	R	R	R	R	R	R	R	R	R	R	R	R	neg	pos	neg	neg	neg	PDR
UCRKP70	no	no	R	R	R	R	R	R	R	R	R	R	R	R	R	R	R	S	neg	pos	neg	neg	neg	XDR
UCRKP71	no	yes	R	R	R	R	R	R	ND	R	R	S	S	S	R	R	R	S	neg	neg	pos	neg	neg	XDR
UCRKP72	no	yes	R	R	R	R	R	R	ND	R	R	S	S	S	S	R	R	S	neg	neg	pos	neg	neg	XDR
UCRKP73	no	yes	R	R	R	R	R	R	S	R	R	I	I	S	R	R	R	S	neg	neg	pos	neg	neg	XDR
UCRKP74	no	no	R	R	R	R	R	R	S	R	R	S	S	S	S	S	R	S	pos	neg	neg	neg	neg	MDR
UCRKP75	no	no	R	R	R	R	R	R	S	R	R	S	R	R	R	R	R	S	neg	neg	pos	neg	neg	XDR
UCRKP76	no	no	R	R	R	R	R	R	ND	R	R	S	R	R	S	R	R	S	neg	neg	pos	pos	neg	XDR
UCRKP77	no	no	R	R	R	R	R	R	S	R	R	R	I	R	R	R	R	S	neg	neg	pos	pos	neg	XDR
UCRKP80	no	no	R	R	R	R	R	R	ND	R	R	S	S	R	R	R	R	S	neg	neg	pos	neg	neg	XDR
UCRKP81	no	no	R	R	R	R	R	R	R	R	R	R	R	R	R	R	R	S	neg	neg	pos	neg	neg	XDR
UCRKP82	yes	no	R	R	R	R	R	R	ND	R	R	R	R	S	R	R	S	S	neg	neg	pos	pos	neg	XDR
UCRKP83	no	no	R	R	R	R	R	R	S	R	R	R	R	R	R	R	R	R	neg	neg	pos	neg	neg	XDR
UCRKP85	yes	yes	R	R	R	R	R	R	S	R	R	S	R	R	R	R	R	S	neg	neg	pos	pos	neg	XDR
UCRKP86	no	yes	R	R	R	R	R	R	S	R	R	R	I	R	R	R	R	S	neg	neg	pos	neg	neg	XDR
UCRKP88	no	no	R	R	R	R	R	R	ND	R	R	R	R	ND	R	R	R	S	neg	neg	pos	neg	neg	XDR
UCRKP89	no	yes	R	R	R	R	R	R	ND	R	R	R	R	R	R	R	R	S	neg	neg	pos	pos	neg	XDR
UCRKP91	yes	no	R	R	R	R	R	R	S	R	R	S	I	S	S	R	S	S	neg	neg	pos	pos	neg	MDR

UCRKP93	no	no	R	R	R	R	R	R	R	R	R	I	R	R	R	R	R	S	neg	neg	pos	neg	neg	XDR
UCRKP94	yes	yes	R	R	R	R	R	R	ND	R	R	I	R	I	R	R	R	S	neg	neg	pos	pos	neg	XDR
UCRKP95	yes	no	R	R	R	R	R	R	ND	R	R	R	R	R	R	R	R	S	neg	neg	pos	neg	neg	XDR
UCRKP97	no	no	R	R	R	R	R	R	ND	R	R	I	I	R	R	R	R	S	neg	neg	pos	pos	neg	XDR
UCRKP99	yes	yes	R	R	R	R	R	R	R	R	R	I	R	R	R	R	R	S	neg	neg	pos	neg	neg	XDR
UCRKP100	yes	yes	R	R	R	R	R	R	S	R	R	R	R	R	R	R	R	S	neg	neg	pos	pos	neg	XDR
UCRKP102	no	yes	R	R	R	R	R	R	S	R	R	S	S	S	R	R	S	S	neg	neg	pos	neg	neg	MDR
UCRKP103	yes	yes	R	R	R	R	R	R	S	R	R	I	R	R	R	R	R	S	neg	neg	pos	neg	neg	XDR
UCRKP104	yes	yes	R	R	R	R	R	R	S	R	R	I	S	S	R	R	R	S	neg	neg	pos	neg	neg	XDR
UCRKP106	yes	no	R	R	R	R	R	R	ND	R	R	R	R	R	R	R	R	R	neg	neg	pos	neg	neg	PDR
UCRKP107	yes	yes	R	R	R	R	R	R	S	R	R	S	S	S	S	R	R	S	neg	neg	pos	pos	neg	MDR
UCRKP108	no	no	R	R	R	R	R	R	R	R	R	R	R	R	R	R	R	S	neg	neg	pos	neg	neg	XDR
UCRKP109	no	yes	R	R	R	R	R	R	S	R	R	S	S	S	R	S	R	S	neg	neg	pos	pos	neg	MDR
UCRKP110	yes	yes	R	R	R	R	R	R	S	R	R	R	R	R	R	R	R	S	neg	neg	pos	pos	neg	XDR
UCRKP111	yes	no	R	R	R	R	R	R	R	R	R	R	R	R	R	R	R	S	neg	pos	pos	pos	neg	XDR
UCRKP112	yes	no	R	R	R	R	R	R	S	R	R	I	R	R	R	R	S	S	neg	neg	pos	neg	neg	MDR
UCRKP113	yes	no	R	R	R	R	R	R	R	R	R	R	S	R	R	R	R	S	neg	pos	pos	pos	neg	XDR
UCRKP114	yes	no	R	R	R	R	R	R	R	R	R	R	R	R	R	R	R	R	neg	neg	pos	neg	neg	PDR
UCRKP115	yes	no	R	R	R	R	R	R	S	R	R	R	R	R	R	R	R	S	neg	neg	pos	neg	neg	XDR
UCRKP118	no	yes	R	R	R	R	R	R	ND	R	R	R	R	R	R	R	R	R	neg	neg	pos	neg	neg	XDR
UCRKP119	yes	yes	R	R	R	R	R	R	S	R	R	R	R	I	R	R	R	S	neg	neg	pos	pos	neg	XDR
UCRKP120	yes	yes	R	R	R	R	R	R	S	R	R	R	R	R	R	R	R	S	neg	neg	pos	pos	neg	XDR
UCRKP121	yes	no	R	R	R	R	R	R	R	R	R	R	R	R	R	R	R	S	neg	pos	pos	neg	neg	XDR
UCRKP122	no	no	R	R	R	R	R	R	R	R	R	I	R	R	R	R	R	S	neg	neg	pos	neg	neg	XDR
UCRKP123	yes	no	R	R	R	R	R	R	S	R	R	S	S	S	R	R	R	S	neg	neg	pos	neg	neg	XDR
UCRKP124	no	yes	R	R	R	R	R	R	ND	R	R	R	S	R	R	R	R	S	neg	pos	pos	pos	neg	XDR
UCRKP125	no	no	R	R	R	R	R	R	R	R	R	R	R	R	R	R	R	S	neg	neg	pos	neg	neg	XDR
UCRKP126	yes	no	R	R	R	R	R	R	S	R	R	R	R	R	R	R	R	S	neg	neg	pos	neg	neg	XDR
UCRKP127	yes	yes	R	R	R	R	R	R	ND	R	R	S	S	S	R	R	R	S	neg	neg	pos	neg	neg	XDR
UCRKP128	no	no	R	R	R	R	R	R	ND	R	R	I	I	R	R	R	R	S	neg	pos	pos	neg	neg	XDR

UCRKP129	yes	no	R	R	R	R	R	R	ND	R	R	R	R	R	R	R	R	S	neg	neg	pos	neg	neg	XDR
UCRKP130	yes	no	R	R	R	R	R	R	S	R	R	R	R	R	R	R	R	S	neg	neg	pos	pos	neg	XDR
UCRKP131	yes	yes	R	R	R	R	R	R	S	ND	ND	S	S	ND	ND	ND	ND	S	neg	neg	pos	neg	neg	MDR
UCRKP132	no	no	R	R	R	R	R	R	S	R	R	R	R	S	R	R	R	R	neg	neg	pos	neg	neg	XDR
UCRKP134	yes	no	R	R	R	R	R	R	S	R	R	I	R	S	S	R	R	S	neg	neg	pos	pos	neg	MDR
UCRKP135	yes	yes	R	R	R	R	R	R	S	R	R	R	R	R	R	R	R	R	neg	neg	pos	pos	neg	XDR
UCRKP136	yes	no	R	R	R	R	R	R	ND	R	R	I	R	S	R	R	R	S	neg	neg	pos	neg	neg	XDR
UCRKP137	yes	yes	R	R	R	R	R	R	ND	R	R	I	R	S	R	R	R	S	neg	neg	pos	neg	neg	XDR
UCRKP138	yes	no	R	R	R	R	R	R	ND	R	R	S	S	S	R	R	R	S	neg	neg	pos	neg	neg	XDR
UCRKP139	yes	no	R	R	R	R	R	R	S	R	R	R	R	S	S	R	R	S	neg	neg	pos	pos	neg	MDR
UCRKP140	yes	yes	R	R	R	R	R	R	S	R	R	R	R	S	R	R	R	S	neg	neg	pos	pos	neg	XDR
UCRKP141	yes	yes	R	R	R	R	R	R	ND	R	R	S	R	R	R	R	R	S	neg	neg	pos	pos	neg	XDR
UCRKP142	yes	yes	R	R	R	R	R	R	S	R	R	S	I	R	R	R	R	S	neg	neg	pos	pos	neg	XDR
UCRKP143	no	no	R	R	R	R	R	R	S	R	R	I	I	R	R	R	R	S	neg	neg	pos	pos	neg	XDR
UCRKP144	yes	no	R	R	R	R	R	R	S	R	R	I	R	R	R	R	R	S	neg	neg	pos	pos	neg	XDR
UCRKP145	yes	yes	R	R	R	R	R	R	S	R	R	I	R	R	R	R	R	S	neg	neg	pos	pos	neg	XDR
UCRKP146	yes	no	R	R	R	R	R	R	ND	R	R	S	I	S	R	R	R	S	neg	neg	pos	pos	neg	XDR
UCRKP147	yes	no	R	R	R	R	R	R	R	R	R	R	R	R	R	R	R	S	neg	neg	pos	neg	neg	XDR
UCRKP149	yes	yes	R	R	R	R	R	R	S	R	R	I	S	S	R	R	R	S	neg	neg	pos	pos	neg	XDR
UCRKP150	no	yes	R	R	R	R	R	R	S	R	S	S	S	R	R	R	R	S	neg	neg	pos	pos	neg	XDR
UCRKP151	yes	yes	R	R	R	R	R	R	S	R	R	S	R	S	S	R	R	S	neg	neg	pos	pos	neg	MDR
UCRKP152	yes	no	R	R	R	R	R	R	S	R	R	S	S	R	R	R	R	S	neg	neg	pos	pos	neg	XDR
UCRKP153	yes	no	R	R	R	R	R	R	S	R	R	S	R	R	R	R	R	S	neg	neg	pos	neg	neg	XDR
UCRKP155	yes	yes	R	R	R	R	R	R	ND	R	R	R	R	S	S	R	R	R	neg	neg	pos	pos	neg	XDR
UCRKP156	yes	yes	R	R	R	R	R	R	ND	R	R	R	R	S	S	R	R	S	neg	neg	pos	pos	neg	XDR
UCRKP157	yes	yes	R	R	R	R	R	R	ND	R	R	I	I	R	R	R	R	S	neg	neg	pos	neg	neg	XDR
UCRKP158	yes	yes	R	R	R	R	R	R	S	R	R	S	S	S	R	R	R	S	neg	neg	pos	neg	neg	XDR
UCRKP159	yes	no	R	R	R	R	R	R	ND	R	R	S	S	S	R	R	R	S	pos	neg	neg	neg	neg	XDR
UCRKP161	yes	yes	R	R	R	R	R	R	S	R	R	R	R	R	R	R	R	S	neg	neg	pos	pos	neg	XDR
UCRKP162	yes	yes	R	R	R	R	R	R	S	R	R	S	I	R	R	R	R	S	neg	neg	pos	pos	neg	XDR

UCRKP163	no	no	R	R	R	R	R	R	S	R	R	I	I	R	R	R	R	S	neg	neg	pos	neg	neg	XDR
UCRKP164	no	no	R	R	R	R	R	R	ND	R	R	R	I	R	R	R	R	S	neg	neg	pos	neg	neg	XDR
UCRKP165	yes	yes	R	R	R	R	R	R	ND	R	R	R	R	R	R	R	R	S	neg	neg	pos	neg	neg	XDR
UCRKP166	yes	yes	R	R	R	R	R	R	ND	R	R	R	R	S	R	R	R	S	neg	neg	pos	neg	neg	XDR
UCRKP167	yes	no	R	R	R	R	R	R	S	R	R	S	S	S	S	R	R	S	neg	neg	pos	neg	neg	MDR
UCRKP168	no	no	R	R	R	R	R	R	S	R	R	I	R	S	R	R	R	S	neg	neg	pos	neg	neg	XDR
UCRKP169	yes	yes	R	R	R	R	R	R	S	R	R	R	R	R	R	R	R	S	neg	neg	pos	pos	neg	XDR
UCRKP170	yes	yes	R	R	R	R	R	R	ND	R	R	S	R	S	R	R	R	S	neg	neg	pos	neg	neg	XDR
UCRKP171	no	yes	R	R	R	R	R	R	S	R	R	S	S	R	R	R	R	S	neg	neg	pos	pos	neg	XDR
UCRKP172	yes	yes	R	R	R	R	R	R	S	R	R	R	R	S	R	R	R	S	neg	neg	pos	neg	neg	XDR
UCRKP173	no	no	R	R	R	R	R	R	R	R	R	R	R	R	R	R	R	S	neg	neg	pos	neg	neg	XDR
UCRKP174	no	no	R	R	R	R	R	R	R	R	R	R	R	R	R	R	R	S	neg	neg	pos	neg	neg	XDR
UCRKP175	yes	yes	R	R	R	R	R	R	S	R	R	R	R	R	R	R	R	S	neg	neg	pos	pos	neg	XDR
UCRKP176	yes	yes	R	R	R	R	R	R	S	R	R	S	I	R	R	R	R	S	neg	neg	pos	pos	neg	XDR
UCRKP177	no	no	R	R	R	R	R	R	S	R	R	I	I	S	R	R	R	S	neg	neg	pos	neg	neg	XDR
UCRKP178	no	no	R	R	R	R	R	R	R	R	R	R	R	R	R	R	R	S	neg	neg	pos	neg	neg	XDR
UCRKP179	no	yes	R	R	R	R	R	R	ND	R	R	R	R	R	R	R	R	S	neg	neg	pos	neg	neg	XDR
UCRKP180	yes	yes	R	R	R	R	R	R	S	R	R	S	R	I	S	R	R	R	neg	neg	pos	pos	neg	XDR
UCRKP181	yes	yes	R	R	R	R	R	R	ND	R	R	S	R	S	R	R	R	S	neg	neg	pos	pos	neg	XDR
UCRKP182	no	yes	R	R	R	R	R	R	ND	ND	R	I	R	S	R	R	R	S	neg	neg	pos	neg	neg	XDR
UCRKP183	yes	yes	R	R	R	R	R	R	S	R	R	S	R	R	R	R	R	S	neg	neg	pos	pos	neg	XDR
UCRKP184	yes	yes	R	R	R	R	R	R	S	R	R	I	R	R	R	R	R	S	neg	neg	pos	pos	neg	XDR
UCRKP185	no	yes	R	R	R	R	R	R	S	R	R	S	S	S	R	R	R	R	neg	neg	pos	neg	neg	XDR
UCRKP 186	yes	no	R	R	R	R	R	R	R	R	R	I	R	R	R	R	R	R	neg	pos	pos	neg	neg	PDR
UCRKP 187	yes	yes	R	R	R	R	R	R	S	R	R	S	R	S	R	R	R	S	neg	neg	pos	pos	neg	XDR
UCRKP 188	yes	yes	R	R	R	R	R	R	S	R	R	I	R	S	R	R	R	S	neg	neg	pos	pos	neg	XDR
UCRKP 189	yes	no	R	R	R	R	R	R	R	R	R	R	R	R	R	R	R	S	neg	pos	pos	neg	neg	XDR
UCRKP 190	yes	no	R	R	R	R	R	R	R	R	R	I	R	R	R	R	R	S	neg	neg	pos	neg	neg	XDR
UCRKP 191	yes	no	R	R	R	R	R	R	S	R	R	S	S	S	S	R	R	S	pos	neg	pos	pos	neg	MDR
UCRKP 192	yes	yes	R	R	R	R	R	R	S	R	R	R	I	S	R	R	R	S	neg	neg	pos	pos	neg	XDR

UCRKP 193	no	yes	R	R	R	R	R	R	R	R	R	R	S	R	R	R	R	S	neg	neg	pos	pos	neg	XDR
UCRKP 194	yes	yes	R	R	R	R	R	R	ND	R	R	R	R	R	R	R	R	S	neg	neg	pos	pos	neg	XDR
UCRKP 195	no	no	R	R	R	R	R	R	ND	R	R	R	R	R	R	R	R	R	neg	pos	pos	pos	neg	PDR
UCRKP 196	no	no	R	R	R	R	R	R	R	R	R	R	R	R	R	R	R	S	neg	pos	pos	pos	neg	XDR
UCRKP 198	no	no	R	R	R	R	R	R	R	R	R	R	R	R	R	R	R	S	neg	pos	pos	pos	neg	XDR
UCRKP 199	yes	no	R	R	R	R	R	ND	ND	ND	R	S	I	R	R	R	R	S	neg	neg	pos	pos	neg	XDR
UCRKP 200	no	no	R	R	R	R	R	R	R	R	R	R	R	R	R	R	R	S	neg	neg	pos	pos	neg	XDR
UCRKP 201	no	no	R	R	R	R	R	ND	ND	ND	R	R	R	R	R	R	R	S	neg	pos	pos	neg	neg	XDR
UCRKP 202	yes	yes	R	R	R	R	R	R	ND	R	R	I	R	R	R	R	R	S	neg	neg	pos	pos	neg	XDR
UCRKP 203	no	yes	R	R	R	R	R	R	ND	R	R	S	S	S	R	R	S	S	neg	neg	pos	pos	neg	XDR
UCRKP 204	yes	yes	R	R	R	R	R	R	R	R	R	R	R	R	R	R	R	R	neg	pos	pos	pos	neg	PDR
UCRKP 205	yes	yes	R	R	R	R	R	R	S	R	R	I	R	S	S	R	R	S	neg	neg	pos	pos	neg	MDR
UCRKP 207	no	no	R	R	R	R	R	R	R	R	R	R	R	R	R	R	R	S	neg	pos	pos	neg	neg	XDR
UCRKP 208	yes	no	R	R	R	R	R	R	S	R	R	S	R	S	R	R	R	S	neg	neg	pos	pos	neg	XDR
UCRKP 209	no	no	R	R	R	R	R	R	ND	R	R	I	R	R	R	R	R	S	neg	pos	pos	pos	neg	XDR
UCRKP 211	yes	yes	R	R	R	R	R	R	S	R	R	S	I	R	R	R	R	S	neg	pos	pos	pos	neg	XDR
UCRKP 212	yes	yes	R	R	R	R	R	R	S	R	R	I	R	S	R	R	R	S	neg	neg	pos	neg	neg	XDR
UCRKP 213	no	yes	R	R	R	R	R	R	S	R	R	R	R	R	R	R	R	S	neg	neg	pos	pos	neg	XDR
UCRKP 214	yes	no	R	R	R	R	R	R	S	R	R	S	S	R	R	R	R	S	neg	neg	pos	neg	neg	XDR
UCRKP 215	no	no	R	R	R	R	R	R	S	R	R	R	R	R	R	R	R	S	neg	neg	pos	neg	neg	XDR
UCRKP 216	no	yes	R	R	R	R	R	R	S	R	R	S	S	R	R	R	R	S	neg	neg	pos	pos	neg	XDR
UCRKP 218	no	no	R	R	R	R	R	R	R	R	R	S	I	R	R	R	R	S	pos	neg	neg	neg	neg	XDR
UCRKP 219	no	no	R	R	R	R	R	R	R	R	R	R	R	R	R	R	R	S	neg	pos	pos	neg	neg	XDR
UCRKP 220	no	no	R	R	R	R	R	R	R	R	R	R	R	R	S	R	R	S	neg	pos	pos	neg	neg	XDR
UCRKP 221	no	no	R	R	R	R	R	R	R	R	R	R	R	R	R	R	R	S	neg	pos	pos	neg	neg	XDR
UCRKP 222	no	no	R	R	R	R	R	R	R	R	R	R	R	R	R	R	R	S	neg	pos	pos	neg	neg	XDR
UCRKP 223	no	no	R	R	R	R	R	R	R	R	R	R	R	R	R	R	R	S	neg	pos	pos	neg	neg	XDR
UCRKP 224	no	no	R	R	R	R	R	R	S	R	R	S	S	S	R	R	R	S	neg	neg	neg	neg	pos	XDR

Resistant (R), Susceptible (S), Intermediate (I), Not determined (ND), multidrug resistant (MDR), pandrug resistant (PDR), extensively drug resistant (XDR).
